# Aggressive prolactinomas responsive to temozolomide treatment: Report of two cases

**DOI:** 10.1002/ccr3.6087

**Published:** 2022-07-18

**Authors:** Zahra Davoudi, Mohammad Hallajnejad, Elena Jamali, Mohammadjavad Honarvar

**Affiliations:** ^1^ Department of Endocrinology Skull Base Research Center of Shahid Beheshti University of Medical Sciences Tehran Iran; ^2^ Department of Neurosurgery Skull Base Research Center of Shahid Beheshti University of Medical Sciences Tehran Iran; ^3^ Department of Pathology, Loghman Hakim Medical Center Shahid Beheshti University of Medical Sciences Tehran Iran; ^4^ Endocrine Research Center, Research Institute for Endocrine Sciences Shahid Beheshti University of Medical Sciences Tehran Iran

**Keywords:** aggressive prolactinoma, case report, pituitary neoplasms, prolactinoma, temozolomide

## Abstract

Refractory aggressive prolactinomas are detected after the unresponsiveness to conventional therapies. We report two cases that underwent temozolomide treatment and have been in near‐complete remission ever since. We suggest the pathology techniques for earlier detection and, subsequently, treatment with temozolomide to reduce morbidities and better respond to therapy.

## Graphical abstract

Patients with refractory aggressive prolactinomas are diagnosed after failure of usual treatments. Since they can have near‐complete remission after treatment with Temozolomide, earlier diagnosis with pathology and treatment can avoid morbidities.

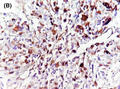

## 1 BACKGROUND

Prolactin‐secreting adenomas constitutes 40% of pituitary adenomas, and about half of prolactinomas are reported to be macroadenomas. Most macroprolactinomas are resolved with conventional therapies with dopamine agonists (such as cabergoline), surgery, and radiation.^1^, ^2^ In 10%–20% of cases, the tumors are unresponsive to conventional therapies.^3^ Previous studies showed that dopamine agonist‐resistant prolactinoma might happen due to decreased dopamine two receptor (DR2) expression in tumor cells.^4^ Aggressive pituitary tumors show various clinical manifestations as they have a tendency to grow, frequently recur, and are often irresponsive to standard treatments.^5^

European Society of Endocrinology guidelines suggest temozolomide (TMZ) monotherapy to be applied after the failure of conventional therapies. TMZ has been shown to shrink pituitary tumors and normalize hormone hypersecretion; thus, TMZ can play an important role in aggressive pituitary adenomas treatment.^6^ The present study reports two cases of macroprolactinomas unresponsive to conventional therapies that underwent therapy with TMZ and have achieved significant tumor regression and clinical improvement.

## 2 CASE PRESENTATIONS

### 2.1 Case 1

**FIGURE 1:**
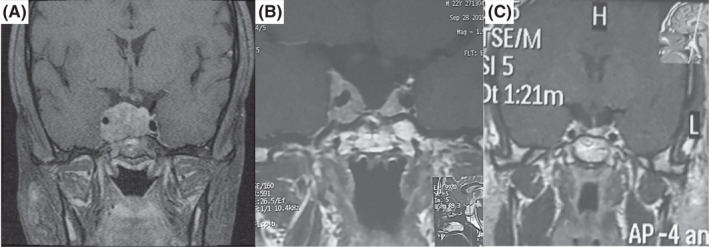
(A) Coronal images of pituitary gland MRI, T1‐weighted post‐contrast demonstrate sellar mass with suprasellar and right cavernous sinus extension. (B) Post‐second surgery and radiation study shows a marked decrease in mass size with residual tumor along the cavernous sinus. (C) After TMZ treatment , there is a near‐complete response

A 22‐year‐old man attended a neurosurgery outpatient clinic with headaches, blurred vision, decreased libido, and ptosis in the right eye. Initial laboratory results were as follows: Luteinizing hormone and follicle‐stimulating hormone serum level of 4 mIU/ml (normal range < 9mIU/L), thyroid‐stimulating hormone of 0.9 mU/L (reference range: 0.5–4.5 mU/L), FT4 of 0.8 ng/dl, (reference range: 0.8–1.8 ng/dl), prolactin (PRL) of >200 ng/ml (reference range < 20 ng/ml), insulin‐like growth factor‐1 of 175 ng/ml (reference range:182–780 mcg/ml), testosterone of 48 ng/dl (reference range:300–1100 ng/dl), adrenocorticotrophic hormone (ACTH) of 17 pg/ml (reference range:10–60 pg/ml), and serum basal cortisol level of 11 μg/dl (reference: 5–25 μg/dl). Prolactin laboratory results are shown in Table 1. MRI showed a macroadenoma with extension to the suprasellar, right cavernous sinus region (KnospIV). Hormonal examination and imaging workups confirmed the diagnosis of a macroprolactinoma (Figure 1A).

**TABLE 1:** PRL levels for two cases before and after surgery and TMZ therapy

	Before 1st surgery	After 1st surgery	After 2nd surgery & radiotherapy	After TMZ therapy	Now
Case 1	>4500 mIU/L (>211.5 ng/ml)	5319 mIU/L (250 ng/ml)	2021 mIU/L (95 ng/ml)	<213 mIU/L (<10 ng/ml)	<213 mIU/L (<10 ng/ml)
Case 2	>5000 mIU/L (>235 ng/ml)	2808 mIU/L (132 ng/ml)	2425 mIU/L (114 ng/ml)	<213 mIU/L (<10 ng/ml)	<213 mIU/L (<10 ng/ml)

**FIGURE 2:**
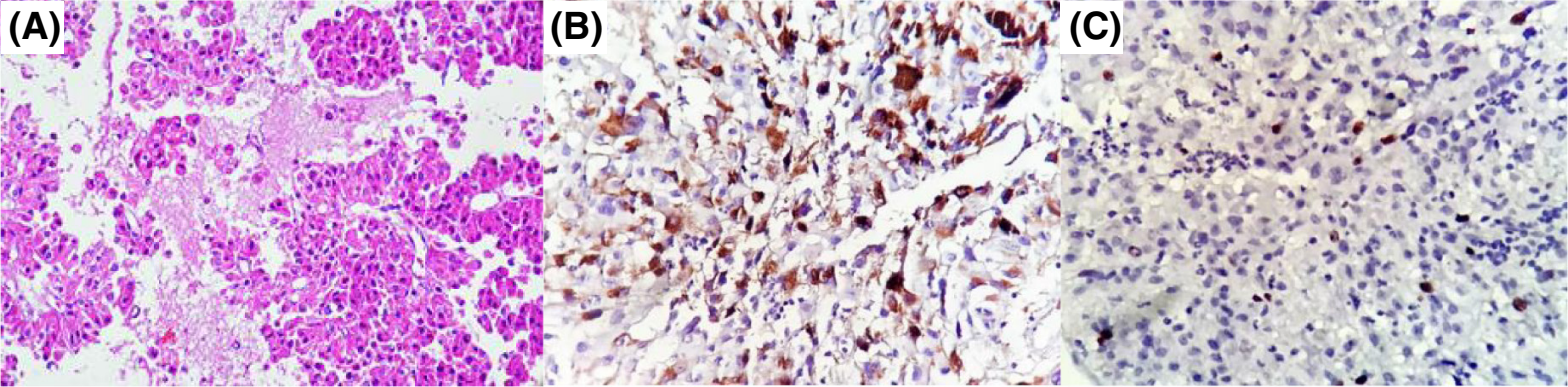
(A) Monomorphic eosinophilic cells of prolactinoma. (B) H&E show diffuse cytoplasmic immunoreactivity for prolactin. (C) increased Ki‐67 proliferative index on IHC study

He underwent endoscopic transsphenoidal surgery (ETSS) based on the patient's desire (opting for surgery instead of cabergoline therapy as the first line of treatment) and cranial nerve involvement (ptosis), and 24 h post‐operation PRL was 250 ng/ml. The histopathologic examination revealed a densely granulated prolactinoma with increased mitotic activity, Ki‐67 proliferative labeling index of 4% and focal immunoreactivity for P53 (in about 8% of tumor cells) (Figure 2). Other pituitary hormones were negative. He was advised to take cabergoline, and despite surgery and medical therapy with weekly 3.5 mg cabergoline, the patient was unresponsive to the treatment.

According to serial MR scan and apparent residual tumor in the right cavernous sinus region, the patient underwent a second surgery. Subsequently, the patient attended 29 sessions of conventional radiotherapy with post‐operation PRL of 95 ng/ml.

After radiotherapy sessions and treatment with 2 mg weekly cabergoline, PRL was 121 ng/ml. MRI still showed a residual mass with invasion to the right cavernous sinus (Figure 1B). Concerning the persistently high level of PRL and invasion to cavernous sinus despite two‐time surgeries and radiotherapy, diagnosis of aggressive (cabergoline medical resistant) pituitary adenoma was made. Therefore, the patient became a candidate for treatment with TMZ. Furthermore, MGMT (O6‐methylguanine‐DNA‐methyltransferase) quantitative methylation analysis was requested that showed negative results (<5%). The immunohistochemical (IHC) study for estrogen receptor (ER) was negative as well (Figure 2).

The patient underwent three courses of TMZ treatment (150–200 mg/m^2^ for 5 days every 28 days) in 3 months, and PRL level decreased; then, treatment was continued for 6 months. Now, the tumor has remarkably shrunk in size (Figure 1c), ptosis resolved, and the PRL level remained in a normal range. The patient is now in a near‐complete remission period with the lowest dose of 0.5 mg weekly cabergoline.

### 2.2 Case 2

**FIGURE 3:**
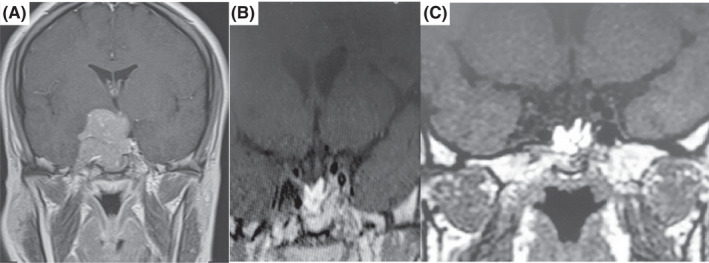
(A) Coronal image of pituitary gland MRI post‐contrast shows large mass obliterating suprasellar cistern with extension into the third ventricular floor, right middle cranial fossa, and right cavernous sinus extension. (B) Post second surgery and radiation study. (C) One‐year post‐TMZ treatment shows a significant decrease in tumor size with a small residual lesion

A 25‐year‐old unmarried man presented with visual disturbances, headache, decreased libido, and weight gain. Pituitary MRI and elevated PRL suggested invasive pituitary macroprolactinoma. As shown in Figure 3A, a 3 × 2 × 2 cm mass was reported in MRI indicative of macroadenoma with invasion in the suprasellar, sphenoidal, and cavernous sinus region (KnospIV). PRL more than 200 ng/ml was reported. With an initial diagnosis of macroprolactinoma, the patient underwent treatment with 3.5 mg weekly cabergoline. After four months, no decrease in PRL level nor tumor size was achieved, so transsphenoidal surgery was performed as the next step.

**FIGURE 4:**
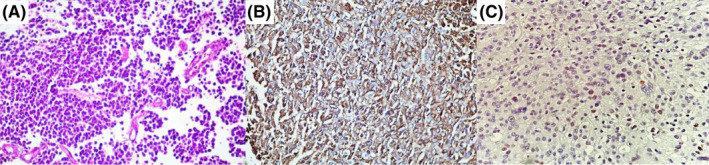
(A) Prolactinoma with monomorphic small cells harboring shrunken cytoplasms. (B) H&E show diffuse cytoplasmic immunoreactivity for prolactin. (C) diffuse weak nuclear staining for estrogen receptoron IHC study

PRL after the first surgery was 132 ng/ml (Table 1). On histopathologic evaluation, densely granulated prolactinoma with Ki‐67 proliferative labeling index of 3% and positive P53 (strong immunoreactivity in 20% of tumor cells) was reported (Figure 4). There was no immunoreactivity for other pituitary hormones in the IHC study. The patient developed panhypopituitarism and diabetes insipidus and was treated accordingly.

During the follow‐up, PRL level was not decreased despite cabergoline therapy (2 mg/weekly) and according to residual tumor in cavernous sinus; the patient underwent a second surgery, and subsequently, the patient attended 29 sessions of conventional radiotherapy with post‐operation and radition PRL of 114 ng/ml (Table 1).

Furthermore, MGMT quantitative methylation analysis was requested that showed negative results (<5%). In the IHC study for ER, diffuse weak immunoreactivity was observed in tumor cells (Figure 4). Therefore, the patient became a candidate for treatment with TMZ with the diagnosis of aggressive pituitary adenoma. Three courses of TMZ treatment (150–200 mg/m^2^ for 5 days every 28 days in 3 months) led to a decrease in PRL level; then, treatment continued for 6 months. As shown in Figure 3c, the last MRI showed tumor regression, and the PRL level remained in the normal range. The patient's visual complaints were resolved. The patient is now (one year after completing TMZ treatment) in near‐complete remission on 0.5 mg weekly cabergoline.

## 3 DISCUSSION AND CONCLUSIONS

Most prolactinomas are benign and responsive to medical therapy. However, in the case of aggressive prolactinomas, due to recurrence and local invasion of these tumors, multiple approaches such as medical therapy, surgery, radiotherapy, and chemotherapeutic agents will be required.^7^ TMZ is an oral antineoplastic alkylating agent firstly used for glioblastoma multiforme. Not long ago, it was found effective in treating refractory aggressive prolactinomas. It is an oral medication, relatively well‐tolerated, and has shown promising results.^8^ Both cases presented in this study had undergone multimodal therapies, which were ineffective as no resolution was observed in laboratory or radiologic studies. The weekly multidisciplinary team meeting held in the tertiary center of the skull base and pituitary surgery decided on the initiation of TMZ for both cases. The optimal duration of TMZ treatment is unknown, and most patients (like the two cases we presented) undergo 6–12 months of treatment, with some centers opting for very long or open‐ended treatment courses.^9^

The shortest duration of treatment with TMZ belongs to Strowd et al.^10^, with three courses of treatment in which recurrence occurred after 2.5 years. Our cases underwent 9 courses, and the cases presented by Ceccato et al.^11^ and Barkhoudarian et al.^12^ underwent 13 and 11 cycles of treatment, respectively. In all these cases who underwent more than six courses of TMZ and had significant reduction in tumor size and prolactin level after the first three courses of TMZ treatment, recurrence did not occur. In the report by Losa et al., of the five cases with aggressive macroprolactinomas, four did not respond and experienced regrowth despite TMZ continuation, while in the case with response to TMZ, recurrence did not occur.^13^

This study used IHC staining to investigate the tumor characteristics. Although we did not have access to a DR2, pathology studies indicated high mitotic index, Ki67 > 3%, positive PS3, densely granulated cells, and low expression of MGMT. The invasion of the tumor to the cavernous sinus and resistance to medical therapy, in addition to the pathologic studies mentioned above indicated that the tumor was invasive and aggressive.^14^ The present cases demonstrated a deficient MGMT protein expression with a near‐complete response to TMZ treatment even one year after stopping it. In previous studies, the effectiveness of TMZ in treating aggressive pituitary tumors showed to be inversely correlated with the expression levels of MGMT.^6^, ^15^, ^16^, ^17^ In addition, McCormack et al.^16^ suggested that hormonal activity, low MGMT expression, and radiotherapy were associated with better outcomes for treating aggressive pituitary adenomas with TMZ, which was consistent with this study's results.

In our cases, the pathology of the tumors was associated with poor estrogen expression. The significant lactotroph regulators are estradiol and dopamine, which play an important role in the regulation and control of cell proliferation and PRL secretion.^17^ In prolactin‐secreting‐adenomas, the primary transcription factors are ERα and pituitary transcription factor 1 (PIT1).^18^

Mahboobifard et al.^19^ have shown that low expression of ERα was accompanied by higher Ki67 indices and showed more invasion properties. They presented two probable mechanisms for ERs downregulation. The first mechanism was the downregulation of ERs secondary to the high activity of estrogen, and the second was the lower ERs expression leading to a decrease in estrogen‐induced apoptosis of lactotroph cells.

Since, unlike multiple previous therapies, these patients responded significantly to treatment with TMZ, the need for a well‐established protocol for aggressive pituitary adenoma or earlier use of this agent in this respect can be helpful. The present study results obtained in these two cases require further research in this field.

## AUTHOR CONTRIBUTIONS

Zahra Davoudi has made major contributions to the manuscript's conception, writing, and revision. Mohammad Hallajnejad was a significant contributor in conception, writing and revising the manuscript. Elena Jamali performed histological examination and contributed to writing the manuscript. Mohammadjavad Honarvar has contributed to acquiring the data and drafting and revising the manuscript. All authors have read and approved the manuscript.

## CONFLICT OF INTEREST

The authors declare that they have no competing interests.

## ETHICAL APPROVAL

This study is according to the ethical principles of the Helsinki Declaration.

## CONSENT

Written consent for publication of the patients' data was obtained from the patients.

## Data Availability

Not applicable.
